# New Three-Dimensional Poly(decanediol-*co*-tricarballylate) Elastomeric Fibrous Mesh Fabricated by Photoreactive Electrospinning for Cardiac Tissue Engineering Applications

**DOI:** 10.3390/polym10040455

**Published:** 2018-04-19

**Authors:** Hesham M. Ismail, Somayeh Zamani, Mohamed A. Elrayess, Wael Kafienah, Husam M. Younes

**Affiliations:** 1 Pharmaceutics & Polymeric Drug Delivery Research Laboratory, College of Pharmacy, Qatar University, P.O. Box 2713, Doha, Qatar; hisma049@uottawa.ca (H.M.I.); zamani@cornell.edu (S.Z.); 2 Anti-Doping Lab Qatar, Sports City Road, P.O. Box 2713, Doha, Qatar; melrayess@adlqatar.qa; 3 Faculty of Biomedical Sciences, School of Cellular and Molecular Medicine, University of Bristol, Bristol BS8 1TH, UK; W.Z.Kafienah@bristol.ac.uk.; 4 Office of Vice President for Research & Graduate Studies, Qatar University, P.O. Box 2713, Doha, Qatar

**Keywords:** poly(diol-tricarballylate), reactive electrospinning, particulate leaching, photo-crosslinking, cardiac tissue engineering, biocompatibility

## Abstract

Reactive electrospinning is capable of efficiently producing in situ crosslinked scaffolds resembling the natural extracellular matrix with tunable characteristics. In this study, we aimed to synthesize, characterize, and investigate the in vitro cytocompatibility of electrospun fibers of acrylated poly(1,10-decanediol-*co*-tricarballylate) copolymer prepared utilizing the photoreactive electrospinning process with ultraviolet radiation for crosslinking, to be used for cardiac tissue engineering applications. Chemical, thermal, and morphological characterization confirmed the successful synthesis of the polymer used for production of the electrospun fibrous scaffolds with more than 70% porosity. Mechanical testing confirmed the elastomeric nature of the fibers required to withstand cardiac contraction and relaxation. The cell viability assay showed no significant cytotoxicity of the fibers on cultured cardiomyoblasts and the cell-scaffolds interaction study showed a significant increase in cell attachment and growth on the electrospun fibers compared to the reference. This data suggests that the newly synthesized fibrous scaffold constitutes a promising candidate for cardiac tissue engineering applications.

## 1. Introduction

Myocardial Infarction (MI) affects almost one million patients yearly, with a development rate to heart failure of more than 30% of the cases in the United States. Current conventional therapies such as coronary angioplasty, cardiac transplantation, and thrombolytic drugs either require complex surgery or finding an appropriate donor, while thrombolytic therapy is not efficient in almost half of the treated population [1]. Indirect immobilization of stem cells and endomyocardial injection were examples of recent alternative regenerative medicine approaches. However, the former was reported to have no effect on the enhancement of the ventricular function over the placebo group after MI, while the latter has exhibited variable results and the optimum route of delivery of stem cells to a specific site remains undetermined [2,3].

Tissue engineering (TE) using biomaterial scaffolds currently emerged as a promising treatment strategy by providing cells with a favorable environment for proper growth and proliferation in a function resembling that of the extra cellular matrix (ECM) [4]. In order to serve this purpose, the ideal scaffold should be hydrophilic with a three-dimensional (3D) highly porous structure, biocompatible, biodegradable, and possess the needed mechanical properties to allow response to different signaling pathways and maintain the strength needed in a mechanically challenging environment [5,6,7]. One more important function to be taken into consideration when developing any scaffold for the regeneration of cardiac tissue is its ability to support the environment capable of possessing the contractile properties of the cardiac tissue and the anisotropic structure of myocardial architecture [8].

Several approaches have been reported to fabricate a porous biomaterial scaffold suitable for TE applications such as: Lyophilization [9], membrane lamination [10], particulate leaching [11], and electrospinning (ES) [12]. Among those techniques, ES was regarded as the most promising fiber production technique capable of producing continuous fibers of a diameter ranging from several micrometers down to 50 nanometers [12]. When the traditional ES was coupled with an in situ crosslinking approach applied to the jet flowing from the needle to the collector, the process was named reactive electrospinning [13]. The in situ crosslinking was either carried out using chemical crosslinkers or by exposing the flowing jet to different types of photo-radiations (e.g., ultraviolet light (UV), laser or gamma radiation), when the process was named photoreactive electrospinning (PRES).

PRES offers numerous advantages over the chemical crosslinking approach. First, efficient and even crosslinking within the scaffold takes place very rapidly at room temperature, in a matter of a few seconds to minutes. Second, it guarantees the production of biocompatible scaffolds as it does not involve the use of toxic chemical crosslinking agents. Although the process involves the use of photo initiators (PI) which are sometimes toxic, the quantity and time of exposure to those PIs during synthesis is negligible and removed later in the process (no detectable traces during characterization). Third, the process may enable the loading and retaining of the stability of growth factors and proteins into the fibers. Finally, the spatial and temporal control can be easily achieved through controlling the exposure area, distance, and the time of light incidence, hence enabling precise control of in situ photocrosslinking, mechanical properties, and elasticity of the fabricated fibers [14]. PRES with UV crosslinking was achieved previously by implementing different strategies. One method was through the addition of photoreactive double bonds to polymers, as in the case of polybutadiene rubber to produce elastomeric fibers [15], commercial polyurethane to produce vascular grafts [16], and methacrylated poly(2,3-dihydroxycarbonate) and polyethyleneimine for TE applications [17,18]. Other double bond sources as cinnamoyl chloride [19] and the azido group in gelatin [20] were also used. 

Attempts to develop the ideal biomimetic scaffold to promote myocardial repair and regeneration are still ongoing. Many previously published studies reported on the utilization of various natural and synthetic polymeric biomaterials and hydrogels to design a suitable scaffold. A large number of these studies focused on using gelatin as a mechanically robust natural polymer which can form a mesh capable of accommodating the contractile properties of the cardiac tissue [21]. Since gelatin degrades relatively rapidly and lacks long term mechanical stability [22], the utilization of slower degradable synthetic thermoplastics such as poly(ε-caprolactone) (PCL), poly(lactide-*co*-glycolide), and poly lactides [23], or thermoset elastomers like poly(glycerol-sebacate), has been reported [24]. However, these materials were either tough, lacked the needed elasticity, and involved complex synthesis procedures, or suffered from plastic deformation and structural failures when used in the applications with tissues of high mechanical stress such as the myocardium. Consequently, the challenge remains to develop biocompatible porous elastomeric fibrous scaffolds with the appropriate mechanical, chemical, and degradation properties. 

We have previously reported on the successful synthesis, characterization, and in vivo biocompatibility testing of a new family of poly(diol-*co*-tricarballylate) thermoset elastomers for TE and drug delivery applications [25,26,27,28]. Herein, we hypothesize that the acrylated poly(1,10-decanediol-*co*-tricarballylate) (APDET) in situ photocrosslinked mesh electrospun fibers (ESF) could act as a cellular niche suitable for cell loading to repair the diseased myocardium. The produced ESF shall address the previously reported drawbacks in compatibility, degradability, and mechanical properties. It would also enable the loading of growth factors and proteins as a result of the use of mild in situ photopolymerization conditions in its fabrication compared to the harsh chemical crosslinking approaches previously reported. As such, in this work, we aimed to synthesize and characterize both the poly(decanediol-*co*-tricarballylate) (PDET) prepolymer and the acrylated form and utilize PRES to produce an elastomeric ESF via optimized parameters. Cell viability and cell-scaffold interaction assessment were performed using H9C2 cardiomyoblasts. PCL-based fabricated fibers were used as a standard reference. Conventional particulate leaching technique using sodium chloride as a porogen was also utilized to produce porous scaffolds for comparison purposes. 

## 2. Materials and Methods

### 2.1. Materials

Tricarballylic acid (TCA), 1,10-decanediol, stannous 2-ethylhexanoate, Triethyl amine (TEA), 2,2-dimethoxy-2-phenylacetophenone (DMPA) and Polyvinylpyrrolidone (PVP), absolute Ethanol, 4-dimethylamino pyridine (DMAP), and phenolphthalein were all purchased from Sigma-Aldrich Chemie GmbH, Taufkirchen, Germany. Acryloyl chloride (ACRL) Lichrosolv^®^ Acetone, Acetic anhydride, Pyridine, Hydrochloric acid (37%), and Chloroform were purchased from Merck, Kenilworth, NJ, USA. All chemicals were used as received without any further purification. Fetal bovine serum, l-Glutamine 200 mM (100×), 2-Mercaptoethanol (50 mM), and the Live/Dead Calcein-AM^®^ Viability/Cytotoxicity assay kit for mammalian cells, Dulbecco modified eagle’s medium (DMEM), and the Gibco^®^ F-12 nutrient mixture were purchased from Life Technologies Co., Paisley, UK. Penicillin-streptomycin and Dulbecco’s phosphate buffered saline (DPBS) were purchased from Sigma-Aldrich Chemie GmbH, Taufkirchen, Germany; DAPI (4′,6-diamidino-2-phenylindole) for nucleic acid staining was purchased from Thermo-Fisher scientific, Waltham, MA, USA. Lonza Trypsin/EDTA (10×) was purchased from SLS life sciences Co., Warwick, UK. They were all used without further purification. 

### 2.2. Methods

#### 2.2.1. Acrylated Poly(decanediol-*co*-tricarballylate) Polymer Preparation

APDET pre-polymer with low and high molecular weight variations and elastomer synthesis, characterization, and end group analysis were carried out as previously reported [25,27]. To synthesize the acrylated polymer, 6 g of PDET pre-polymer was dissolved in 60 mL acetone, to which 6 mg DMAP was added as a catalyst and placed in a 250 mL round-bottom flask equipped with a magnetic stirrer. The flask was sealed, flushed with argon, and then immersed in a 0 °C ice bath. A stepwise addition of 1.6 mL ACRL (19.8 mmol) with an equimolar amount of TEA to the pre-polymer solution was carried out over a period of 12 h. The equivalent molar amount of triethylamine was used to scavenge the HCl formed during the reaction. The reaction was later continued at room temperature for another 12 h. The reaction completion was detected using thin layer chromatographic analysis and the final solution was filtered to remove TEA salt formed during the acrylation reaction. The acrylated polymer solution was then dried at 45 °C under vacuum (63.5 mmHg) using a rotary evaporator. 

#### 2.2.2. Scaffold Production by Conventional Particulate Leaching Process

Sodium chloride was sieved into a defined particle size (250 µm < X < 500 µm) using Retsch AS200 (Retsch, Haan, Germany) sieves. APDET was added in an Eppendorf tube with 1 µL of photo initiator (2,2-dimethoxy-2-phenylacetophenone in acetone, 20% *w*/*v*) and mixed by a spatula, followed by the addition of NaCl (70% *w*/*w*). Ingredients were mixed again until a paste was formed. The whole mixture was transferred into the middle of a petri dish. Afterwards, the cover of the petri dish was placed upside down to spread the paste into a disc. The petri dish containing the disc paste was placed under UV light of 100 watts at a 365 nm wavelength (Blak-Ray, UVP-LLC, Upland, CA, USA) for 10 min for each side of the dish for homogenous and even crosslinking. Afterwards, the upper cover was removed slowly, followed by filling the petri dish with deionized water for 5 min, and the disc was removed slowly by a spatula. A blank 2D scaffold was prepared using the same procedure but without the addition of NaCl to be used as a control. Scaffolds were soaked in 1500 mL of deionized water (Milli-Q Direct, Millipore, Burlington, MA, USA) for four days with mild stirring. The scaffolds were then allowed to dry under the hood for 24 h.

#### 2.2.3. Electrospun Fibrous Mesh Production by Electrospinning of the Acrylated Polymer

The electrospinning solution was prepared by dissolving APDET in ethanol in a sealed glass vial using a magnetic stirrer until a clear solution was obtained. This was followed by the addition of PVP with continuous stirring until the solution became clear again. The ideal preparation composition was found to be 20% *w*/*v* APDET, 8% *w*/*v* PVP 130 kDa in ethanol. Afterwards, 0.1% (*w*/*w*) of the photoinitiator, 2,2-dimethoxy-2-phenylacetophenone was added and allowed to dissolve. The solution was transferred into a syringe supplied with an electrospinning needle of size 23 G. The syringe was placed in a syringe pump (NaBond, Shenzhen, China). The needle was then connected to one of the electrodes of a power supply that generated the voltage difference between the needle and the grounded plane aluminum plate collector. Finally, in situ crosslinking was performed by placing a UV lamp of 100 watts (Neo pro, NaBond, Shenzhen, China) at a distance of 10 cm from the polymer jet. The parameters of the electrospinning process used were as follows: Voltage difference of 17–22 KV, tip to collector distance (TCD) of 18 cm, the UV lamp was placed 10 cm away from the polymer jet, and the flow rate was 10 mL/h for 30 min. The ESF were kept under UV for an extra 10 min after electrospinning to ensure full crosslinking of the polymer. 

#### 2.2.4. Chemical and Thermal Characterization

Fourier transform infrared (FT-IR) and Proton Nuclear Magnetic Resonance (^1^H-NMR) analyses were carried out on prepolymer, acrylated polymers, PVP, and ESF as reported earlier [25]. Molecular weight and molecular weight distribution of the synthesized pre-polymer were determined using the Viscotek GPC max VE2001 gel permeation chromatography system (GPC) (Viscotek, Malvern Instruments, Malvern, UK). The GPC max was equipped with triple detector array TDA 305 multiple detection composed of a refractive index (RI) detector, right angle and low angle laser light scattering detectors (RALS/LALS), a viscometer, and a UV detector. The column setup was composed of a general purpose aqueous column set of a single pore size column (T6000, org GPC/SEC col, 300 × 8 mm, exclusion limit 20,000 kDa), two single pore size columns (T1000, Org GPC/SEC col, 300 × 8 mm, exclusion limit 1500 Da), and one Flow injection polymer analysis (FIPA) non-polar organic column (H100-3078, 100 × 7.8 mm). The molecular weights were determined using a dn/dc of 0.954 mL/g calculated from the RI detector response and assuming 100% mass recovery through the column. As for the mobile phase, the polymer sample was dissolved in analytical grade acetone at a concentration of 20 mg/mL. The flow rate was 1 mL/min and the injection volume was 100 μL at 35 °C. The data was collected and analyzed using the OmniSEC 5.02 software package (Malvern Panalytical Ltd., Malvern, UK. Thermal properties of the prepolymer, PVP powder, and PVP/APDET ESF were analyzed using Differential Scanning Calorimetry (DSC-8000) (PerkinElmer, Billerica, MA, USA) equipped with a liquid nitrogen intracooling system (Intracooler II). The heating rate was 10 °C/min. Samples were cooled down to −70 °C and then heated up to 300 °C to record the DSC thermograms. 

#### 2.2.5. Scanning Electron Microscopy, Fiber Diameter, and Pore Size Analysis

Scanning Electron Microscope (SEM) (Bruker, Billerica, MA, USA) and Nikon eclipse (LV100NPOL) polarizing microscope (Nikon, Tokyo, Japan) were used to examine the structure and surface morphology of both the ESF and the disc scaffolds produced by the particulate leaching method. SEM was performed on gold sputter coated samples to improve the sample conductivity. Polarizing microscope was also used, especially during the in process monitoring of ES. A glass slide was placed on the collector and left for one minute to allow the ESF to deposit on the slide. The slide was then placed under the microscope and a series of magnifications was used to obtain the results. Average fiber diameter and pore size were calculated using the NIH approved ImageJ software (National Institute of Mental Health, Bethesda, MD, USA) for image analysis. The calculation was performed by measuring 25 fibers and pores respectively. Results were reported as mean ± standard error of mean (SEOM) (*n* = 3).

#### 2.2.6. Porosity Calculations

The porosity of the produced scaffold was calculated using the liquid intrusion method. The weight of the dry scaffold or ESF was measured and it was then placed in ethanol for 24 h. Afterwards, the scaffold’s surface was wiped gently to remove excess ethanol and was then weighed. Porosity (P) was calculated using the following equation:

P = V_ETH_/(V_ETH_ + V_APDET_)

where V_ETH_ is the volume of ethanol absorbed by the scaffold into the pores. It was calculated by dividing the change in scaffold mass after immersion in ethanol and the density of ethanol (0.789 g/cm^3^). V_APDET_ is the volume of the dry scaffold fibers and it was calculated as the ratio between the weight of the dry scaffold and the density of TCA.

#### 2.2.7. Tensile Testing and Contact Angle Measurements

Tensile testing was done using the Instron 3343 single column table frame (Instron Co., Norwood, MA, USA) equipped with a 1 N load cell, as reported earlier [25]. The ESF were cut into dog-bone shaped specimens and placed between the grips of the machine at room temperature. The samples were pulled at a rate of 1 mm/s until breaking. Mean ± standard deviation, percent elongation, and young’s modulus were calculated using Bluehill management software using *n* = 5. Young’s modulus was calculated from the initial slope of the stress-strain curve. The crosslinking density was calculated according to the theory of rubber elasticity using the equation: *ρ*x = E/3RT, where *ρ*x is the number of active network chain segments per unit volume (mol/m^3^), E is the young modulus in Pascal, R is the universal gas constant (R = 8.314 J/mol K), and T is the absolute temperature in kelvin. PCL electrospun fibers were used as a reference for comparison purposes. 

The contact angle for the ESF was measured using the DSA25 goniometer (Krüss GmbH, Hamburg, Germany) provided with a microsyringe with a PTFE needle with a diameter of 0.5 mm. The drop shape was analyzed using the sessile drop method on a drop of purified water, 10 mL in volume dispensed from the PTEF needle. After the drop and the mesh surface came into contact, a digital camera captured the drop shape in 5 s, and the drop contour was mathematically represented by the Young Laplace equation. The contact angle was calculated as the slope of the contour line at the three-phase contact point. Five measurements were taken for different sites and the data was represented as mean ± SEOM.

#### 2.2.8. In Vitro Cytocompatibility Studies

##### Cell Isolation and Seeding

H9C2 myoblasts were obtained from the European Collections of Cell Cultures (ECACC) (Salisbury, UK) and cultured according to the manufacturer’s instructions. Cells were seeded in 48-well plates (BD Falcon) at a seeding density of 30,000 cells/well in 300 μL of media. Cells were incubated and allowed to attach for 24 h before scaffold addition. ESF/Scaffolds were cut as discs using biopsy punches with a unified diameter and were sterilized by immersion in 70% ethanol for 10 min, followed by washing three times with PBS solution to remove any traces of ethanol. Scaffolds/ESF were incubated with cells for 24 and 48 h. 

##### Quantitative Cell Viability Assay

Cells were fixed in 4% formaldehyde, stained with DAPI, and scanned using ArrayScan™ XTI (Thermo Fisher Scientific, Waltham, MA, USA). The number of viable cells was assessed by automated quantitation of DAPI positive nuclei using the target activation module, following exclusion of apoptotic nuclei distinguished by their collapsed size, higher chromatin intensities, and nuclear fragmentation [29]. Images were taken for 20 fields per well and analysis and cell counting was performed by HCS Studio™ Cell Analysis Software (Thermo-Fisher scientific, Waltham, MA, USA). 

##### Cell/Scaffold Interaction

Sterile scaffolds/ESF were placed in ultra-low attachment (non-tissue culture treated) (Thermo-Fisher scientific, Waltham, MA, USA) plates. Cells were added directly to the scaffolds/ESF at a seeding density of 1 × 10^4^ cells/scaffold and were allowed to attach for 2 h under 5% CO_2_/air atmosphere at 37 °C. Afterwards, appropriate volumes of culture media were added to the wells and changed every other day for a total of 14 days. At the end of the incubation period, viable cells attached to the scaffolds were stained using the Calcein-AM from the LIVE/DEAD^®^ viability/cytotoxicity assay. Fluorescent images were taken using an inverted microscope with a monochrome digital camera using 5× magnification (Carl Zeiss Micro-Imaging, New York, NY, USA). Cell viability was assessed visually by estimating cell-covered areas compared to the control.

#### 2.2.9. Statistical Analysis

All values were reported as mean ± SEOM. Statistical analysis was done using an independent *t*-test for fiber diameter and pore size analysis and ANOVA for cell viability using SPSS software (IBM, Armonk, NY, USA). Results were considered statistically significant at *p* < 0.05.

## 3. Results and Discussion

**Figure 1:**
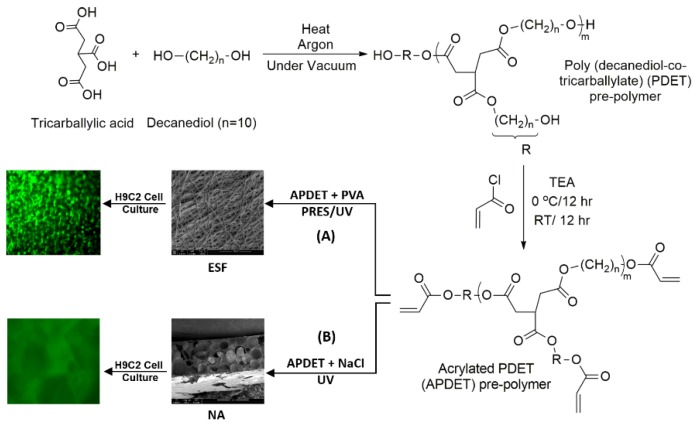
Schematic illustration of the PDET synthesis, acrylation, and further crosslinking to prepare (A) electrospun fibers (ESF) using UV-based photoreactive electrospinning (PRES) or (B) UV photocrosslinked scaffolds using the NaCl particulate leaching technique (NA).

Our main aim was to fabricate ESF from the APDET prepolymer utilizing UV light through PRES. A schematic illustration showing the PDET synthesis, acrylation, and further crosslinking to prepare the ESF or the photocrosslinked scaffolds using NaCl as a porogen is shown in Figure 1. First, the pre-polymer was synthesized from tricarballylic acid and excess decanediol via the polycondensation reaction. The reaction was carried out under argon followed by vacuum to remove the produced water. To synthesize the low and high molecular weight variations of the pre-polymer, we diversified the reaction temperature (130–160 °C), the argon interval (10–45 min), and the vacuum interval (35–75 min). The optimized reaction with the best viscous yet flowing pre-polymer was achieved at a temperature range of 140–150 °C, argon interval of 20 min, and vacuum interval of 50 min. When the temperature, argon, or vacuum intervals were increased slightly above these conditions, the polymer changed into its rubbery state due to extensive thermal crosslinking. The produced pre-polymer was of a faint yellow color. The pre-polymer was then dissolved in chloroform followed by filtration to remove any excess unreacted residues. The second step involved the acrylation of the pre-polymer where the terminal hydroxyl groups were converted into vinyl groups to prepare it for further crosslinking. Both the acrylated and the non-acrylated preparations were soluble in most organic solvents as ethanol, chloroform, acetone, and methanol, but formed a colloidal dispersion with water that was white in color.

### 3.1. Chemical & Thermal Characterization

#### 3.1.1. Molecular Weight Measurements

The molecular weight (Mwt) and molecular weight distributions of the pre-polymer using GPC are reported in Table 1. Variations of Mwt have been prepared by varying the conditions of the pre-polymer condensation reaction to change the degree of crosslinking [25]. As expected, the measurements showed that the variation in the reaction conditions changed the Mwt of the pre-polymer. The highest Mwt which the prepolymers reached while still flowing freely was 5190 Da. At that Mwt, the viscous portion was still exceeding the elastic portion and hence, it was maintained at its liquid state. When the Mwt reached a higher value, the prepolymer crosslinked to a rubbery state in which the elastic portion exceeded the viscous portion. Moreover, the extensive reaction led to the consumption of most of the hydroxyl groups, which made the pre-polymer flow poorly and further acrylation could not be performed easily. In addition, end group analysis of both the high and low Mwt variations of the prepolymer was conducted and is reported in Table 1. This was necessary to determine the amount of terminal hydroxyl groups, which corresponded to the optimum amount of acryloyl chloride, needed for subsequent successful acrylation. It was also important to adjust the degree of acrylation and hence to ensure successful crosslinking through the added vinyl groups. In conclusion, the high molecular weight variation of our pre-polymer was the one utilized further in this study.

#### 3.1.2. FT-IR, NMR & DSC Analysis

**Figure 2:**
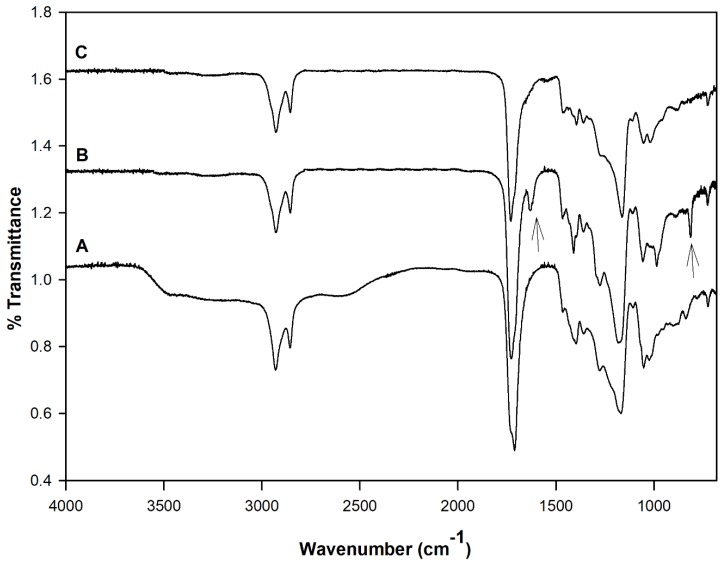
FT-IR analysis of PDET-based polymer (5190 KDa). (A) Before acrylation (PDET), (B) after acrylation (APDET), and (C) after photoreactive electrospinning (crosslinked APDET based electrospun fibers). The two arrows correspond to the CH_2_=CH_2_ bands formed.

Figure 2 shows the FT-IR spectra of the PDET prepolymer, APDET before crosslinking, and the photocrosslinked APDET. As shown in Figure 2A, a broad band appeared at 3600–3400 cm^−1^ that was indicative of the hydroxyl stretching vibrations. The bands at around 2900–2800 cm^−1^ correspond to C–H stretching. The sharp absorption band at 1730 cm^−1^ was attributed to the ester carbonyl group. The bands between 1000–1300 cm^−1^ represent the vibrations of C–O stretching. After acrylation, and as shown in Figure 2B, the broad band at 3600–3400 cm^−1^ corresponding to the hydroxyl group almost disappeared, accompanied by the appearance of two new bands at 1630 & 815 cm^−1^ corresponding to CH_2_=CH_2_. Those two small bands are indicative of the successful incorporation of the acryloyl terminal moieties. However, the band areas were very small due to the consumption of most of the acryloyl moieties during the free radical polymerization reaction when crosslinking occurred (Figure 2C). The FT-IR results corresponded well with those reported earlier [25].

**Figure 3:**
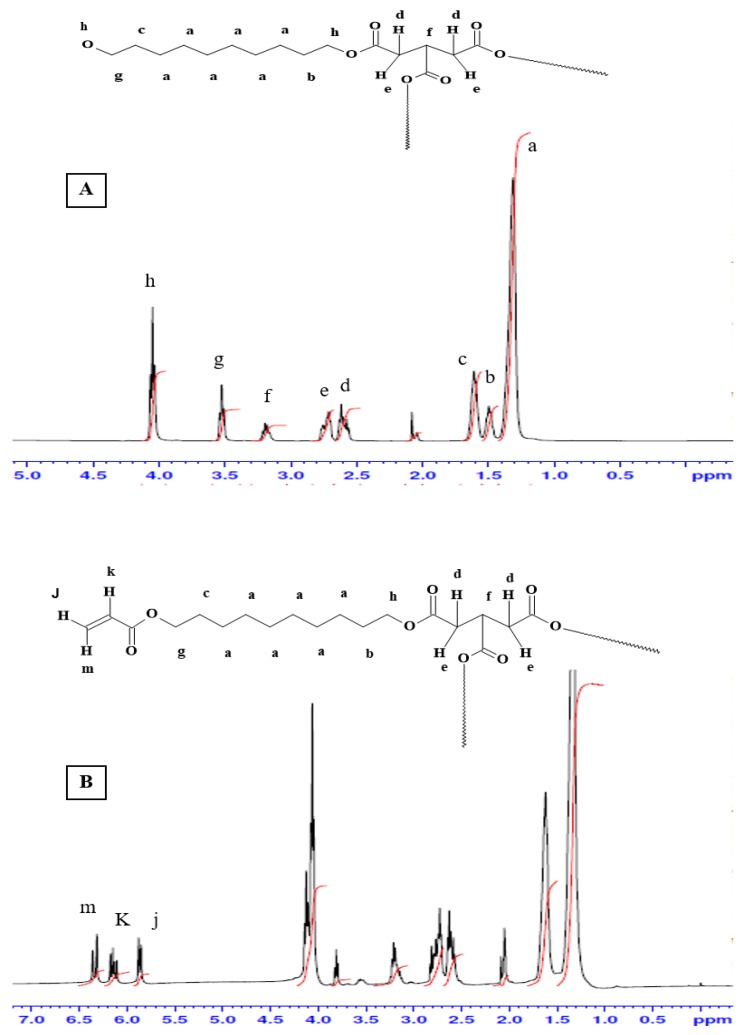
H-NMR Spectra of (**A**) PDET and (**B**) APDET. Black line represents the peaks with their corresponding chemical shifts. Red vertical lines represent the integrals of those peaks.

Figure 3 shows the ^1^H-NMR spectra for the pre-polymer and the acrylated pre-polymer. As shown in the Figure 2A, peak (a) represents protons of the methylene groups of the 1, 10 decanediol that appeared at 1.35 ppm. The protons of the pre-terminal carbon of the diol were represented by peaks (b) and (c) at 1.5 ppm and 1.6 ppm, respectively. Protons next to the pro-chiral center of TCA were embodied by peaks (d) and (e) at 2.65 ppm and 2.75 ppm, respectively, while the peak (f) at 3.2 ppm signified the protons of the pro-chiral center of TCA. As for protons of the diol’s terminal carbon (adjacent to the hydroxyl group) and protons of the carbon attached to the ester bond, they were seen as peaks at 3.5 ppm (g) and 4.05 ppm (h), respectively. The acrylation process was proven successful upon the appearance of the three peaks at 5.8 (j), 6.1 (k), and 6.4 ppm (m), representing the newly introduced vinyl group (Figure 2B).

DSC analysis was performed to analyze the thermal characteristics of PDET and APDET before and after crosslinking, as well as after PRES. This was done to detect the effects of UV crosslinking, as well as electrospinning on the polymer. The PDET prepolymer showed a crystalline structure arrangement with a melting point of −3 °C and a glass transition temperature (Tg) of −36 °C. After acrylation, the vinyl group introduction at the terminals of the prepolymer caused a restriction in the chains’ movement, preventing rearrangement and resulting in an increase in Tg to 26 °C. This restriction was intensified after UV crosslinking due to increased structural stiffness upon the change from liquid state to elastomeric amorphous state. PVP which was reported to have a melting point of around 130 °C showed no melting endotherm as an indication of its conversion to the amorphous form. Consequently, after PRES, the ESF preserved their amorphous structure with no effect, resulting from PVP addition.

### 3.2. Morphological Analysis

Two types of scaffolds were synthesized: The first type was fabricated using the traditional particulate leaching process in which 70% *w*/*w* sodium chloride was used as a porogen. These scaffolds were used as references for comparison purposes. The second type was fabricated using the PRES process to utilize its unique advantages to produce a highly porous ESF for the same purpose.

**Figure 4:**
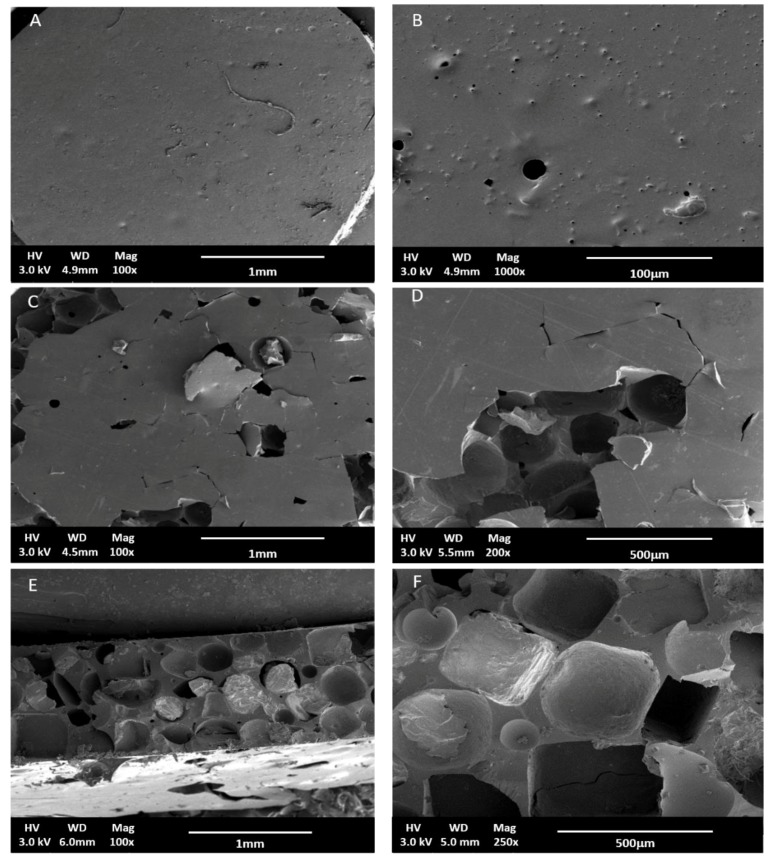
SEM micrographs of APDET scaffolds produced by particulate leaching using NaCl as a porogen. (**A**,**B**) Blank films, (**C**,**D**) Top view of porous APDET scaffolds, and (**E**,**F**) Sectional view of porous APDET scaffolds.

Figure 4 shows SEM images of scaffolds produced by particulate leaching. As shown in the figure, scaffolds produced using NaCl as a porogen were of low surface porosity since it was difficult to leach out all NaCl particles and residual crystals existed within the matrix even after a long leaching out period, as demonstrated in Figure 4E,F. This finding was congruent with what was reported previously [30]. The inner part of the scaffolds and the surface of all scaffolds were porous compared to the blank films. However, one of the disadvantages of particulate leaching is that the size of the interconnected pores is hard to control. Nonetheless, due to its simplicity, it is widely accepted as a reference for comparison with different scaffold production techniques [31].

### 3.3. Effect of PVP, Polymer Concentration, Molecular Weight, and Acrylation Degree on Fiber Formation

PVP 90 KDa (PVP90) and PVP1300 KDa (PVP1300) were used and each was mixed with different concentrations of APDET pre-polymer. Ethanol was used as the solvent because it was a suitable solubilizer to all components including PVP and it gave the best bead-less fibrous structure during ES with a narrow fiber distribution compared to other organic solvents. It also has a high dielectric constant that aided in the electrospinning process [32,33]. Finally, as Ethanol did not evaporate rapidly, it prevented the breakdown of the polymer jet during ES and hence supported the fiber formation process.

To optimize the ESF formation process, an ideal combination of concentration of APDET/PVP in the solution had to be concluded. The theoretical entanglement concentration for PVP in ethanol was previously calculated to be 7.5% *w*/*v*. However, previous work reported an ideal PVP concentration between 7% and 9% *w*/*v* to form bead-less fibers [33]. In our study, an 8% PVP concentration resulted in the optimum viscosity and bead-less fiber formation.

**Figure 5:**
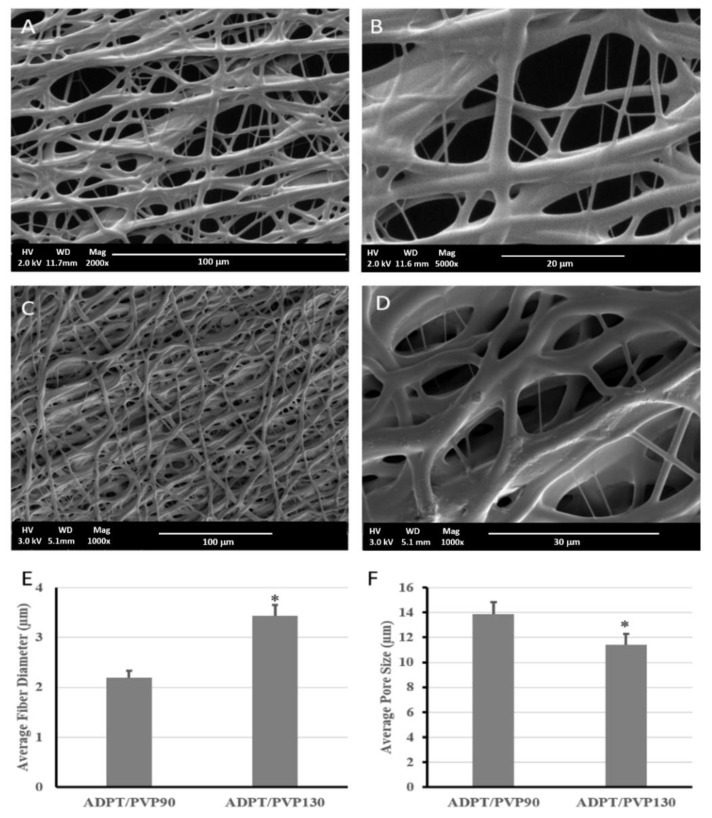
Morphological structure and analysis of ADPT/PVP electrospun fibers with a 2× acrylation degree at different PVP molecular weights. (Upper) SEM micrographs (**A**,**B**) with PVP 90 KDa, (**C**,**D**) with PVP 1300 kDa. (Lower) plot of (**E**) Fiber diameter and (**F**) Average pore size (**E**). Data were represented as mean ± SEOM (*n* = 5), * *p* < 0.05.

The optimum APDET (MWt 5190 Da) concentration that produced a mesh structure was found to be 20% *w*/*v*. Below this concentration, fiber fusion took place. This was attributed to insufficient APDET for a proper degree of crosslinking to prevent fusion. On the other hand, an APDET concentration above 30% *w*/*v* resulted in a very fast crosslinking for the polymer droplet on the tip of the needle, which led to needle clogging. When electrospinning was attempted using PVP90, a porous fibrous mesh was produced, as shown in Figure 5. This was due to the higher acrylation degree, which increased the number of terminal double bonds that elevated the crosslinking degree and hence guaranteed faster, more efficient crosslinking, less fiber fusion, and more structural integrity. Other studies have reported the use of PVP1300 as an entanglement enhancer which aided in the successful formation of ESF [18]. We also attempted using PVP1300 in our experiments, as in Figure 5C,D.

As shown in Figure 5, a porous mesh structure was formed upon using PVP1300, but exhibited a significantly larger fiber diameter and smaller pore size than when PVP90 was used. This resulted from the increased viscosity of the solution as the molecular weight of the used PVP increased [34,35,36]. The ESF produced using PVP1300 was further used for in vitro studies since it was the one used to calculate the critical entanglement concentration reported earlier [37]. All of the produced porous ESF showed secondary fiber branching as demonstrated by the SEM micrographs that rendered our ESF resembling the spider net nanofibers reported earlier [38]. This is due to the branching of the primary polymer stream into several secondary jets due to further instabilities within the straight jet or after Tylor cone [39]. These secondary jets might give rise to beads or fibers depending on their stability upon responding to the applied voltage. If those streams were broken into droplets, beads were formed. On the contrary, in our case, the unbroken secondary polymer jets formed fibers and were deposited on the collector, causing the appearance of those branches of a smaller diameter that stemmed from the main fibers [40,41].

### 3.4. Porosity Analysis

The porosity and pore sizes of the scaffolds fabricated by electrospinning and particulate leaching techniques were compared. All fabricated scaffolds/ESF were highly porous, with porosities exceeding 70%. Pore size analysis revealed a difference between the two scaffolds. Those produced by particulate leaching reached a porosity of 72% and showed the highest pore size (304.8 ± 13.2 µm), although they had less surface pores compared to the latter. On the other hand, the ESF possessed a 78% porosity and showed the lowest pore size among all specimens (11.43 ± 0.78 µm). The importance of the pore size, porosity, and fiber diameter appeared upon the assessment of cells interactions with the scaffold/ESF, where an optimum combination of all factors should facilitate cell attachment and growth in the 3D scaffolds. This is discussed later under the in vitro studies section of this paper.

### 3.5. Tensile Testing

Table 2 summarizes the results of the tensile testing performed on the ESF compared to PCL-based electrospun scaffolds as the control in a dry state. PCL was chosen because it was widely used for the same area of application and is known to be the material of choice for tunable mechanical properties, alone or in combination with other polymers [42]. It was previously reported that mechanical stress enhanced bioengineered cardiomyocyte proliferation, intercellular organization, and cardiomyocyte hypertrophy. The bioengineered human cardiomyocytes could contract autonomously with a proper response to contraction. Moreover, when bioengineered cardiomyocytes were transferred into the hearts of nude rats, they survived, engrafted onto the heart, and were perfused by blood vessels of the host [43]. Consequently, the mechanical properties of the scaffolds should be suitable for physical support of the cells. They should also be flexible, possessing a suitable tensile strength and young’s modulus to withstand and allow the conduction of impulses to the loaded cells through the myocardium. If both of these properties were high, this would cause resistance to the cardiac contraction and might constrain the heart relaxation.

As listed in Table 2, the ESF showed a lower young’s modulus compared to the PCL fibers (control). This indicated their high elasticity, which would enable the scaffold to withstand myocardial contraction and relaxation. In addition, the length of the scaffold before and after breakage was the same, allowing for full recovery upon stretching and exposure to stress cycles. This was due to the elastomeric behavior of the crosslinked polymer that allowed full recovery upon deformation. This was contrary to PCL-based fibers, which deformed after breaking, indicating their inability to retain their original geometry and to consequently suffer from plastic deformation [44]. On the other hand, when our PDET-based ESF were compared to the non-porous photocrosslinked films reported earlier by our group [25], there was an increase in both the tensile strength and young’s modulus. This was attributed to the higher crosslinking degree for the ESF due to the higher energy of UV light than visible light used previously, which meant a higher crosslinking efficiency. This indicated the ease of manipulation of the mechanical properties of our ESF to suit the purpose of use by changing the degree of acrylation or the crosslinking degree upon changing the intensity and time of exposure to UV light. Previous reports indicated a young’s modulus of up to 0.02 MPa and 0.5 MPa at the beginning and end of cardiac diastole of human myocardium, respectively [24]. Consequently, the elastomeric properties and behavior of the fabricated ESF would enable them to withstand the myocardial contraction and relaxation and hence, they could be considered a very promising candidate for cardiac tissue engineering applications [45].

### 3.6. Contact Angle Measurement

Scaffolds used for tissue engineering applications are preferably hydrophilic to allow for the easy attachment of cells in a way resembling ECM. When the contact angle was measured for the prepared ESF, it was found to be 80° ± 5.3, which indicated the moderately hydrophilic nature of those scaffolds. It was previously reported that cells like cardiomyocytes preferably attach to polymers with an intermediate contact angle of around 70° [46]. This indicated that the fabricated ESF would be promising candidates for cardiac tissue regeneration. In addition, when the contact angle was measured at multiple time points, it was found to gradually decrease to a minimum of 30.4°. This might be due to the porous nature of the ESF, together with its hydrophilicity, where the droplet propagates through the scaffold, decreasing the contact angle. This was strong evidence of the hydrophilic nature of our scaffolds.

### 3.7. In Vitro Cell Viability and Cell/Scaffold Interactions

#### 3.7.1. Cell Viability Study Using H9C2 Cardiomyoblasts

**Figure 6:**
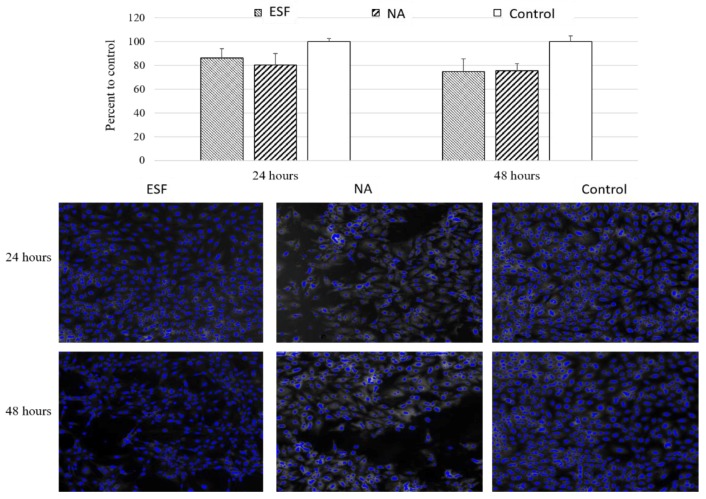
The effect of different synthesized scaffolds on H9C2 cell viability. Cells were incubated with both ESF and NA for 24 and 48 h and cell number was assessed by automated quantitation of DAPI positive nuclei using ArrayScan XTI (Target activation module). (**Upper**) the number of nuclei of viable cells represented as percentages relative to untreated control. Data presented as Mean ± SEOM, *n* = 6. Statistical significance: * *p* < 0.05 compared to the control. (**Lower**) Representative Images of the DAPI stained nuclei of viable cells after incubation with the scaffolds.

The main aim for producing the scaffolds was to utilize their characteristics for cardiac tissue engineering applications. As part of scaffold validation, the effect of synthesized scaffolds on cell viability was verified. For this purpose, the two types of synthesized scaffolds were tested for indirect cytotoxicity due to traces of toxic chemicals used in the original reaction or any potential toxic degradation products using H9C2 cardiomyoblasts. These cells can differentiate into cardiomyocytes by lowering serum levels, and can thus be used for cardiac tissue engineering purposes [47]. As shown in Figure 6, there was no significant cytotoxicity detected following 24 or 48 h of incubation with either scaffolds. The viability compared to the control was 86.2 ± 11.0% for ESF and 80.4% ± 13.7 for sodium chloride particulate leached scaffolds (NA). This data suggested that both scaffolds exhibited no significant cytotoxic effect in vitro and may be considered for tissue engineering purposes pending further in vivo confirmation.

#### 3.7.2. Assessment of Cell/Scaffold Interaction

**Figure 7:**
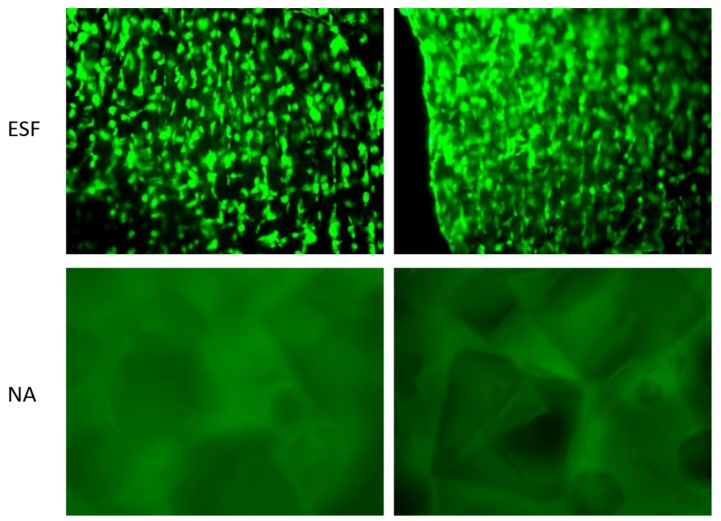
Qualitative assessment of cell/scaffold interaction in non-tissue culture treated plates. H9C2 cells were directly seeded on the scaffolds and incubated for 14 days in non-tissue culture treated plates. At day 14, cells were stained with Calcein-AM and representative images of H9C2 cells on ESF (**top**) and NA (**bottom**) were captured using a fluorescent microscope. Live cells appear as a fluorescent green color.

In order to assess the direct interaction between synthesized scaffolds and cells, H9C2 cardiomyoblasts were seeded on ESF or NA and incubated for 14 days in non-tissue culture treated well plates before being visualized using Calcein-AM staining. As shown in Figure 7, H9C2 cardiomyoblasts successfully attached to ESF, while there was no apparent attachment to the NA scaffolds. Cells were growing on different planes of view within ESF, indicating the penetration of cells through its 3D structure (Figure 7, top). The efficiency of cell attachment to ESF was high with an even distribution, facilitated by the 3D mesh structure that allows the pre-adsorption of serum protein and enhancement of cell attachment, as previously reported [44]. Different cell types have different optimum pore sizes for maximum growth, proliferation, and differentiation [48,49,50,51]. For H9C2 cells, a higher degree of attachment on ESF was attributed to an optimum pore size, pore size distribution, and fiber diameter compared to the NA scaffold. Similar findings using adipose derived human mesenchymal stem cells grown on electrospun scaffolds for 14 days demonstrated the important role of fiber diameter and pore size in the orientation and infiltration of the cells in the scaffold [52]. With this respect, a future study using the ESF over a prolonged period would be promising for other TE applications.

Interestingly, H9C2 cells demonstrated a certain degree of alignment along the fibers (Figure 7), potentially due to the fiber collection method used in this study. Similar aligned structures offering the required anisotropic effect consistent with cardiac tissue engineering applications were previously reported [53]. Such an effect provides a superior advantage over other types of scaffolds as it enhances cardiomyocyte cell attachment with a specific orientation compared to random oriented electrospun scaffolds [53].

## 4. Conclusions

In this study, we successfully synthesized the APDET polymer with a high molecular weight. Afterwards, we successfully produced ECM resembling porous ESF using the promising PRES technique. In addition, we fabricated APDET-based porous scaffolds using the particulate leaching technique and sodium chloride as a porogen. Full chemical, thermal, morphological, and mechanical characterization was performed for all the fabricated scaffolds. Morphological analysis demonstrated a three-dimensional structure with a high porosity and a variable pore size range. In addition, mechanical testing of the ESF demonstrated a high elasticity that was suitable for cardiac tissue engineering purposes. The high crosslinking density of the produced scaffold offered another aspect of the highly tunable mechanical properties of the APDET polymer during synthesis or fabrication. ESF were resistant to different solvents while preserving their fibrous structure. The produced scaffolds were of an amorphous nature with a Tg lower than the core body temperature, as represented by thermal analysis. This would ensure the existence of the scaffold in the rubbery elastic state at all times, which is required to withstand the continuous cardiac contraction and relaxation.

The in vitro quantitative cell viability assay of cardiomyoblasts showed no significant cytotoxic effect when cells were co-cultured with ESF and NA scaffolds. However, ESF demonstrated superior cell attachment and growth over NA when cell scaffold interaction was investigated, suggesting that ESF are promising candidates for cardiac TE applications. Further investigations are warranted to confirm cell-scaffold interaction and their potential therapeutic applications.

## Figures and Tables

**Table 1:** Molecular weights, molecular weight distribution, and the determined hydroxyl end groups of different pre-polymer variations determined via GPC.

Variation (PDET Pre-Polymer)	*M*w	*M*n	*M*w/*M*n	OH (mmol/g) *
Very high molecular weight	6542	6104	1.072	--
High molecular weight	5190	3657	1.419	1.1
Low molecular weight	3538	1031	3.4	1.6
Very low molecular weight **	1366	1090	1.25	2.7

* Determined by end group analysis; ** Data were reported previously by our group [25].

**Table 2:** Mechanical properties of different scaffolds/fibers.

Sample/Parameter	UTS (Mpa)	Elongation (%)	Modulus (Mpa)	Crosslinking Density (mol/m^3^)
PCL Fibers (Control)	1.63 ± 0.32	175.43 ± 22.74	2.4 ± 0.79	-
APDET based ESF	0.54 ± 0.24	46.93 ± 11.93	1.35 ± 0.34	168.85 ± 29.43
PDET * 2D scaffolds	0.14 ± 0.02	61.23 ± 5.83	0.33 ± 0.02	44.39 ± 3.09

UTS: Ultimate tensile strength; * Data were reported previously by our group [25].
